# Association of cytokeratin 17 expression with differentiation in oral squamous cell carcinoma

**DOI:** 10.1007/s00432-012-1202-6

**Published:** 2012-04-03

**Authors:** Ryoji Kitamura, Takeshi Toyoshima, Hideaki Tanaka, Shintaro Kawano, Takahiro Kiyosue, Ryota Matsubara, Yuichi Goto, Mitsuhiro Hirano, Kazunari Oobu, Seiji Nakamura

**Affiliations:** Section of Oral and Maxillofacial Oncology, Division of Maxillofacial Diagnostic and Surgical Sciences, Faculty of Dental Science, Kyushu University, 3-1-1 Maidashi, Higashiku, Fukuoka, 812-8582 Japan

**Keywords:** Cytokeratin 17, Oral squamous cell carcinoma, Leukoplakia, Differentiation, Cytokeratin 13, Immunohistochemistry

## Abstract

**Purpose:**

The aim of this study was to confirm the expression profile of cytokeratin (CK)17 in comparison with that of CK13 in oral squamous cell carcinoma (OSCC) and leukoplakia and to clarify an association of CK17 with the OSCC differentiation.

**Materials:**

The expression of CK17 and CK13 was immunohistochemically examined in 105 patients with OSCC and 108 patients with leukoplakia. A correlation of CK expression with clinicopathological variables was carried out. The over-expression levels of CK17 mRNA were analyzed by real-time RT-PCR in 5 OSCC cell lines (HSC-2, HSC-3, SAS, SQUU-A, SQUU-B).

**Results:**

CK17 and CK13 were detected in 101 (96.2 %) and three (2.9 %) of the 105 OSCCs, respectively. CK17 was significantly expressed in well-differentiated OSCC compared to moderately/poorly differentiated OSCC (*p* < 0.01). As detected in 19 of the 34 dysplastic leukoplakias (55.9 %) and 36 of the 74 hyperplastic leukoplakias (48.6 %), CK17 was significantly expressed in dysplastic leukoplakias (*p* < 0.01). As detected in 11 of the 34 dysplastic (32.4 %) and 52 of the 74 hyperplastic leukoplakias (70.3 %), CK13 was significantly expressed in hyperplastic leukoplakias (*p* < 0.01). The relative expression of CK17 mRNA in HSC-2 was significantly higher than in HSC-3 and SAS (*p* < 0.05). Moreover, the relative expression of CK17 mRNA in SQUU-A was significantly higher than in SQUU-B (*p* < 0.05).

**Conclusion:**

CK17 expression could be associated with the differentiation and the malignancy of OSCC. A combination pattern of CK17/CK13 might be a suitable marker of malignant transformation.

## Introduction

Despite diagnostic and therapeutic advances by introducing combination therapy including surgery, radiotherapy, and chemotherapy, the 5-year survival rate of oral squamous cell carcinoma (OSCC) remains 70–80 % (Gorsky et al. 2004; Sasaki et al. 2011), because of the late-stage diagnosis and the resistance to radiotherapy or chemotherapy. For early diagnosis and individual therapy of the patients, it is of great importance to find out diagnostic markers of OSCC. Cytokeratins (CKs), intermediate filament of the cytoskeletons, are candidates for diagnostic markers of OSCC, as they are over-expressed in OSCC compared to normal mucosa (Xu et al. 1995). Biochemical and immunohistochemical studies have indicated that OSCC expresses a wider range of CKs than normal epithelium, because cancerization of normal oral epithelium could lead to variation of differentiation degree (Wetzels et al. 1992).

Among CKs, CK17 should be focused as a diagnostic marker of OSCC, since several studies have reported that the expression of CK17 could be detected in malignant tissues compared to normal tissues in squamous cell carcinoma of lung (Wetzels et al. 1992), cervix (Carrilho et al. 2004; Ikeda et al. 2008), larynx (Cohen-Kerem et al. 2004), and esophagus (Takahashi et al. 1995; Luo et al. 2004). In oral cavity, our previous study based on the microarray and the real-time RT-PCR analyses have reported that the significant up-regulation and the strong over-expression of CK17 could exhibit the utility as diagnostic marker of OSCC (Toyoshima et al. 2008). Focused on differentiation of cells expressing CK17, CK17 is also considered as a marker of basal cell differentiation to distinguish columnar epithelium from squamous epithelium in cervix (Martens et al. 2004). Moreover, CK17 is markedly expressed in well-differentiated squamous cell carcinoma (Carrilho et al. 2004). However, to our knowledge, a paucity of material is available on a correlation between the CK17 expression and the differentiation in OSCC.

CK13 is mucosa specific and expressed in the suprabasal layers of non-keratinized stratified epithelia. In normal tissue, CK13 is usually expressed in the conjunctival and limbal epithelium (Ramirez-Miranda et al. 2011). On the contrary, decreasing of CK13 expression is found in dysplastic lesions in the oral epithelium and associated with the development of OSCC and oral leukoplakia (Ohta et al. 2010). Because of the loss of stratification, CK13 is down-regulated in malignant lesion (Bloor et al. 2000). Carrilho et al. (2004) have reported that the expression of CK17 and the loss of CK13 are appropriate markers of malignant transformation in cervix carcinoma. In addition, Whipple et al. (2004) have reported that CK17 mRNA is up-regulated, while CK13 mRNA is down-regulated in OSCC by the microarray analyses. Moreover, it has been reported that CK17 is significantly up-regulated in leukoplakia and is absent in normal oral epithelium (Mikami et al. 2011). In contrast, CK13 is down-regulated in leukoplakia and firmly expressed in normal oral epithelium. However, little is known about an association of CK17 and CK13 expressions with the differentiation of OSCC and leukoplakia.

Therefore, the aim of this study was to confirm the immunohistochemical profile of CK17 in comparison with that of CK13 in OSCC, leukoplakia, and normal oral epithelium from a differentiation point of view. Secondly, we focused on CK17 mRNA expression in OSCC cell lines in order to clarify an association of CK17 with the differentiation of OSCC.

## Materials and methods

### Patients

One hundred and five patients with primary OSCC and 108 patients with primary leukoplakia, diagnosed at the Department of Oral and Maxillofacial Surgery, Kyushu University Hospital, from 2005 to 2008, were enrolled in this study. Ten control cases (normal oral epithelium) were also enrolled. Patients’ informed consent and the approval of the local ethical committee were given. Following the initial biopsy, all specimens were fixed in 4 % buffered formalin solution and embedded in paraffin blocks. Subsequently, the paraffin-embedded specimens were processed to 5-μm-thick sections, stained with hematoxylin–eosin (HE), and examined by three experienced pathologists to confirm the diagnoses and the histological grades. The histological grade in the OSCC and the degree of epithelial dysplasia in the leukoplakia were assessed according to the World Health Organization classification (Gale et al. 2005; Wahi et al. 1977). The tumor extent and the clinical stages were evaluated according to the TNM classification established by the American Joint Committee on Cancer and the International Union Against Cancer (UICC) (Sobin and Wittekind 2002). The mode of invasion was also determined on the HE-stained specimens according to Yamamoto-Kohama’s criteria, as follows: grade 1 = well-defined borderline; grade 2 = cords, less-marked borderline; grade 3 = group of cells, no distinct borderline; grade 4 = diffuse invasion, 4C: cord-like type; 4D: widespread type (Yamamoto et al. 1984). Medical records were reviewed to collect the information concerning the clinical characteristic. The detailed clinical data of the patients with OSCC and leukoplakia are presented in Tables 1 and 2.

**Table 1:** Characteristics of 105 patients with OSCC

Characteristics	Category	Cases (%)
Gender	Male	53 (50.5)
	Female	52 (49.5)
Age	65>	61 (61.0)
	65<	44 (39.0)
Alcohol	Yes	56 (53.3)
	No	49 (46.7)
Smoking	Yes	48 (45.7)
	No	57 (52.3)
Differentiation	Well	72 (68.6)
	Moderate	31 (29.5)
	Poor	2 (1.9)
T	1	41 (39.0)
	2	47 (44.8)
	3	3 (2.9)
	4	14 (13.3)
N	Positive	20 (19.0)
	Negative	85 (81.0)
Mode of invasion	1	9 (8.6)
	2	21 (20.0)
	3	49 (45.7)
	4C	19 (18.1)
	4D	7 (6.7)
Clinical stage	I	42 (40.0)
	II	33 (31.4)
	III	8 (7.6)
	IV	22 (21.0)
Localization of the lesion	Tongue	63 (60.0)
	Gingiva	23 (21.9)
	Buccal mucosa	8 (7.6)
	Floor of mouth	7 (6.7)
	Palate	4 (3.8)

**Table 2:** Characteristics of 108 patients with leukoplakia

Characteristics	Category	Cases (%)
Gender	Male	68 (63.2)
	Female	40 (36.8)
Age	65>	51 (48.1)
	65<	57 (53.8)
Degree of epithelial dysplasia	Hyperplasia	74 (69.8)
	Mild	20 (18.9)
	Moderate	8 (7.5)
	Severe	6 (5.7)
Localization of the lesion	Gingiva	45 (42.5)
	Tongue	44 (41.5)
	Palate	10 (9.4)
	Buccal mucosa	7 (6.6)
	Lip	1 (0.9)
	Floor of mouth	1 (0.9)

### Immunohistochemistry

Immunohistochemical staining was performed on 5-μm-thick sections sliced serially from paraffin-embedded blocks after formalin fixation of the excised specimens. The sections were deparaffinized in xylene and rehydrated in a graded series of ethanol/water concentrations (100, 95, 90, 85, and 75 %). For antigen retrieval, the sections were immersed in DAKO Target Retrieval Solution (Dako Cytomation, Denmark) and autoclaved at 120 °C for 5 min. The endogenous peroxide activity was then eliminated with 1 % hydrogen peroxide for 30 min, and the section was rinsed twice for 10 min with phosphate-buffered saline (PBS) at pH 7.4. Non-specific protein binding was blocked by incubation for 1 h with 10 % goat serum, and then, the sections were incubated with each primary antibody for 3 h at room temperature. The following primary antibodies were used: anti-human monoclonal CK17 antibody (clone E3, Dako Cytomation, Denmark; diluted 1:60) and anti-human monoclonal CK13 antibody (clone KS-1A3, Novocastra, Germany; diluted 1:1000). The sections were rinsed twice for 10 min with PBS and incubated with secondary antibodies conjugated with peroxidase-labeled amino acid polymer for 1 h at room temperature. After rinsing with PBS twice for 10 min, the immunoreactivity was visualized by immersing the sections in 3, 3′-diaminobenzidine and 0.6 % hydrogen peroxide (DAB substrate kit, Nichirei, Japan). Subsequently, the sections were counterstained with Mayer’s hematoxylin, dehydrated in graded ethanol (75, 85, 90, 95 and 100 %), cleared with xylene, and finally mounted with permanent mounting medium (Mount-Quick, Daido Sangyo, Japan). Negative controls were prepared by substituting PBS for each primary antibody. To evaluate the expression of CK17 and CK13 in OSCC and leukoplakia, positively stained cells were counted in at least three randomly selected areas at ×200 magnifications, and then, each percentage of these positive cells was calculated as a BIOREVO fluorescent microscope (Keyence, Japan). The stained sections with <5 % reactive cells were considered to be negative, and those with more than 5 % reactive cells were defined as positive. The cutoff points were established at 30 and 60 %, and 5–30 %, 30–60 %, and more than 60 % reactive cells defined as +, ++, and +++, respectively. The sections were divided into two groups as follows: over 60 % positive cells were defined as “strong” case; less than 60 % positive cells were defined as “weak” case.

### Cell lines

Five human OSCC cell lines (HSC-2, HSC-3, SQUU-A, SQUU-B, and SAS) were used in the study. Human epithelium cell line (HaCaT) was used as a calibrator. HSC-2 was established from well-differentiated OSCC of mouth floor, while HSC-3 and SAS were established from poorly differentiated OSCC of tongue. SQUU-A and SQUU-B cell lines were established from well-differentiated OSCC of tongue with local recurrences. Cells were maintained in Dulbecco’s modified Eagle’s minimum essential medium (DMEM) supplemented with 10 % fetal bovine serum (FBS) and incubated at 37 °C in a 5 % CO_2_ atmosphere. Culture media was changed on alternate days during the experiments.

### Real-time RT-PCR

Total RNA was extracted from these 6 cell lines with the TRIzol^®^ reagent (Invitrogen, USA). cDNAs from total RNA were synthesized using the GeneAmp RNA PCR kit (Applied Biosystems, USA) according to the manufacturer’s instructions. Real-time RT qPCR analyses were done using QuantiTect Primar Assay (200) [Hs_KRT17_1_SG QuantiTect Primer Assay (200) (Cat.QT00001680) for CK17, QIAGEN]. For normalization, GAPDH was used. The relative quantification (RQ) of mRNA was performed with STRATAGENE MX7000. The Brilliant^®^ SYBR^®^ Green QPCR Master Mix (Agilent Technologies, USA) was used for PCR amplification. In total, 40 ng of cDNA was used for each PCR in a total volume 20 μl. Each PCR run included a 15-min activation time at 95 °C as required by the instrument. The three-step cycle included denaturing (94 °C, 15 s), annealing at 55 °C, and extension at 72 °C. At the end of each PCR run, melting curve analysis was performed from 60 to 95 °C for detecting non-specific PCR product and primer–dimer co-amplification. CK17 mRNA quantities were analyzed in duplicate, normalized GAPDH as an internal control gene and expressed in relation to mRNA from HaCaT as a calibrator. Results are expressed as relative gene expression using the ΔΔCt method (Livak and Schmittgen 2001).

### Statistical analysis

In the real-time RT-PCR, mean value of duplicate CK17 mRNA RQs was defined as positive if it was higher than 2.0-fold. The statistical significance of the differences between the groups was determined by Student’s *t*-test and the chi-square test for univariate analyses. The interaction between CK17 expression and the other variables in patients with OSCC was determined by multivariate logistic regression analysis. *p* values of <0.05 were considered to be significant.

## Results

### Immunohistochemical analyses of CK17 and CK13 expression in OSCC

**Fig. 1:**
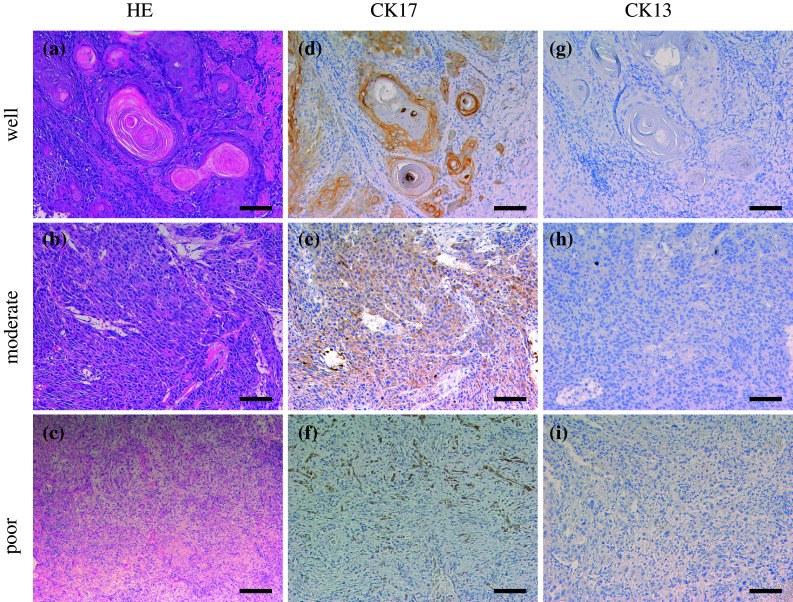
Immunohistochemical staining of CK17 and CK13 in OSCC. Serial sections were stained with HE (**a**, **b**, **c**) anti-CK17 antibody (**d**, **e**, **f**), and anti-CK13 antibody (**g**, **h**, **i**), and each representative case is shown here. In well-differentiated OSCC, CK17 was strongly expressed in the majority of tumor cells and was strongly expressed in the inner of cancer nest (**d**). In moderately differentiated OSCC, CK17 was weakly expressed in the majority of tumor cells (**e**). In poorly differentiated OSCC, CK17 was not expressed in the majority of tumor cells (**f**). CK13 was not expressed in most of OSCC cases (**g**–**i**). (Original magnification ×100, *scale bars* 100 μm)

A representative case of CK17 and CK13 expressions is shown in Fig. 1. CK17 and CK13 were predominantly expressed in the cellular cytoplasm. In well-differentiated OSCC, CK17 was strongly expressed in the majority of tumor cells. In the cancer nest, CK17 was expressed in the inner layers and not expressed in the outer layers (Fig. 1d). In moderately differentiated OSCC, CK17 was weakly expressed in the majority of tumor cells (Fig. 1e). In poorly differentiated OSCC, CK17 was absent in the majority of tumor cells but expressed in a few of tumor cells (Fig. 1f). CK13 was detected in three of the 105 OSCCs (Fig. 1). In these cases, CK13 was expressed in the center of cancer pearls in well-differentiated OSCC. There were no significant differences between CK13 expression and clinicopathological variables (data not shown).

CK17 was detected in 101 of the 105 OSCC cases (96.2 %). The “strong” cases with over 60 % reactive cells were 23 of all 72 well-differentiated OSCCs (31.9 %). The “strong” case was one of all 33 moderately and poorly differentiated OSCCs (3.0 %). CK17 was significantly expressed in well-differentiated OSCC compared to moderately and poorly differentiated OSCC (*p* < 0.01, chi-square test; Table 3), though there were no significant differences for other clinical variables (Table 3). All variables were further analyzed in a multivariate logistic regression model. The result of the analysis showed that the OSCC differentiation was the significant factor of CK17 “strong” cases (*p* < 0.01, multivariate logistic regression analysis; Table 3). Gender, age, alcohol, smoking, TNM classification, and localization of the lesions were not significant (Table 3).

**Table 3:** Uni- and multivariate analyses for association between CK17 expression and clinicopathological variables in 105 patients with OSCC

Variables	CK17 expression^a^		Univariate analysis		Multivariate analysis	
	“Weak”^b^	“Strong”	OR (95% CI)	*p* values	OR (95% CI)	*p* values
Gender						
Male	42	11	Reference	–	Reference	–
Female	39	13	0.786 (0.31–1.96)	0.605	0.97 (0.26–3.47)	0.959
Age						
65>	45	16	Reference	–	Reference	–
65<	36	8	0.63 (0.23–1.59)	0.335	0.63 (0.17–2.27)	0.480
Alcohol						
Yes	41	15	Reference	–	Reference	–
No	38	11	1.14 (0.45–3.02)	0.776	3.26 (0.63–19.23)	0.160
Smoking						
Yes	36	12	Reference	–	Reference	–
No	43	14	0.80 (0.31–2.01)	0.632	0.46 (0.09–2.25)	0.332
Differentiation^c^						
Well	49	23	Reference	–	Reference	–
Moderate + Poor	32	1	15.02 (2.93–275.21)	0.009	44.65 (4.94–1786.55)	0.001
T						
1+2	68	20	Reference	–	Reference	–
3+4	13	4	0.95 (0.29–3.68)	0.943	4.75 (0.23–183.39)	0.319
N						
Positive	14	6	Reference	–	Reference	–
Negative	67	18	1.59 (0.51–4.61)	0.401	4.20 (0.17–197.74)	0.386
Mode of invasion^d^						
1	8	1	Reference	–	Reference	–
2	16	5	0.40 (0.02–3.07)	0.437	0.69 (0.03–7.74)	0.783
3	36	13	0.35 (0.02–2.16)	0.339	0.42 (0.02–3.48)	0.456
4C	15	4	0.47 (0.02–3.88)	0.528	0.32 (0.01–5.11)	0.433
4D	6	1	0.75 (0.03–21.63)	0.849	0.47 (0.01–21.05)	0.677
Clinical stage^e^						
I	35	7	Reference	–	Reference	–
II	23	10	0.46 (0.15–1.37)	0.163	0.37 (0.09–1.45)	0.155
III	7	1	1.40 (0.20–28.28)	0.762	1.90 (0.03–320.93)	0.782
IV	16	6	0.53 (0.15–1.90)	0.324	0.60 (0.01–61.35)	0.823
Localization of the lesion						
Tongue	49	14	Reference	–	Reference	–
Gingiva	14	9	0.44 (0.16–1.26)	0.122	0.24 (0.04–1.13)	0.072
Others	18	1	5.14 (0.92–96.50)	0.126	4.87 (0.61–111.50)	0.147

^a^CK17 was detected by immunohistochemical staining, and the degree of expression was estimated as described in the “Materials and Methods”

^b^The number of cases is indicated

^c^The histological grade of differentiation was determined according to the criteria of WHO

^d^The histological grade of tumor invasion was determined according to Yamamoto and Kohama’s criteria

^e^The clinical stages were determined according to the International Union Against Cancer (UICC)

*OR* odds ratio, *CI* confidence interval

**Fig. 2:**
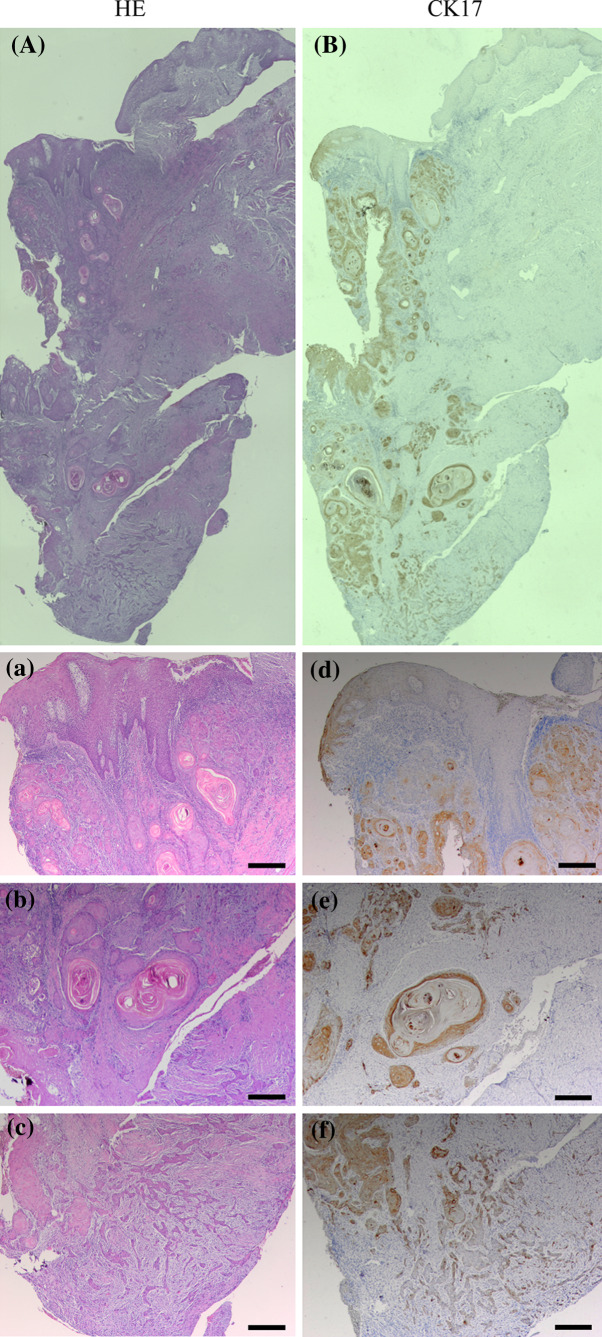
A representative case of OSCC with dysplasia, cancer nests, and infiltrating cells. Serial sections were stained with HE (**A**, **a**–**c**) and anti-CK17 antibody (**B**, **d**–**f**). High-magnification views of selected areas are shown (**a**–**f**). CK17 was markedly expressed in dysplasia, while not in normal epithelium (**d**). CK17 was obviously expressed in the inner layer of cancer nest, while not in the outer (**e**). CK17 was absent in the infiltrating cells (**f**). (Original magnification ×40, *scale bars* 200 μm)

A representative case of CK17 expression in moderately differentiated OSCC is shown in Fig. 2. CK17 was markedly expressed in the dysplastic epithelium adjacent to the tumor, while CK17 was not completely expressed in the normal oral epithelium (Fig. 2d). In the cancer nest, CK17 was obviously expressed except for the outer layer and the center of cancer pearl (Fig. 2e). In the invasion front of the tumor, CK17 was absent in the infiltrating cells (Fig. 2f).

### Immunohistochemical analyses between CK17 and CK13 in leukoplakia and normal oral epithelium

**Fig. 3:**
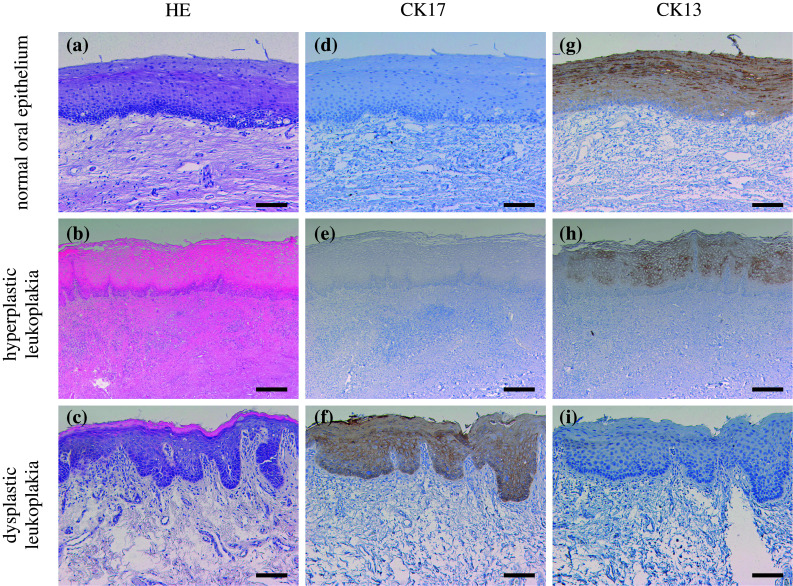
Immunohistochemical staining of CK17 and CK13 in the normal oral epithelium and dysplastic and hyperplastic leukoplakias. Serial sections were stained with HE (**a**–**c**), anti-CK17 antibody (**d**–**f**), and anti-CK13 antibody (**g**–**i**), and each representative case is shown here. CK17 was not expressed in normal oral epithelium and hyperplastic leukoplakia (**d**, **e**). CK17 was markedly expressed (**f**) in dysplastic leukoplakia. CK13 was expressed in normal oral epithelium and hyperplastic leukoplakia (**g**, **h**) and not expressed in dysplastic leukoplakia (**i**). (Original magnification ×100, *scale bars* 100 μm)

CK17 was locally expressed in prickle cell layer of leukoplakia, while it is not expressed in normal oral epithelium (Fig. 3). CK17 was detected in 55 of the 108 leukoplakias (50.9 %). CK17 was detected in 19 of all 34 dysplastic leukoplakias (55.9 %) and 36 of all 74 hyperplastic leukoplakias (non-dysplasia) (48.6 %). The “strong” cases were 8 of the 34 dysplastic leukoplakias (23.5 %); on the other hand, the “strong” cases were 3 of the 74 hyperplastic leukoplakias (4.0 %). CK17 was significantly expressed in dysplastic leukoplakias compared to hyperplastic leukoplakias (*p* < 0.01, chi-square test; Table 4).

**Table 4:** Association between CK17 and CK13 expression and clinicopathological variables in 108 patients with leukoplakia

Variables	Category	Number of cases^b^	CK17 expression in leukoplakia^a^				Significance^c^	CK13 expression in leukoplakia^a^				Significance^c^
			–	+	++	+++		–	+	++	+++	
Gender	Male	68	37	4	17	10		30	18	6	14	
	Female	40	16	3	20	1	NS	15	9	7	9	NS
Age	65>	51	24	5	18	4		24	10	7	10	
	65<	57	29	2	19	7	NS	21	17	6	13	NS
Degree of dysplasia^d^	Non-dysplasia	74	38	7	26	3		22	19	10	23	
	Mild	20	9	0	7	4		14	6	0	0	
	Moderate	8	2	0	3	3		4	2	2	0	
	Severe	6	4	0	1	1	*p* < 0.01	5	0	1	0	*p* < 0.01
Localization of the lesion	Gingiva	45	28	3	14	0		19	10	6	10	
	Tongue	44	20	2	12	10		17	12	5	10	
	Palate	10	1	1	8	0		3	3	2	2	
	Buccal mucosa	7	4	0	2	1		4	2	0	1	
	Lip	1	0	0	1	0		1	0	0	0	
	Floor of mouth	1	0	1	0	0	NS	1	0	0	0	NS

^a^CK17 and CK13 were detected by immunohistochemical staining, and the degree of expression was estimated as described in the “Materials and Methods”

^b^The number of cases is indicated

^c^Statistical significance of the association was determined by the chi-square test

^d^The histological degree of dysplasia was determined according to the criteria of WHO

*NS* not significance

CK13 was expressed in prickle cell layer of the epithelium. CK13 was detected in all 10 normal oral epitheliums (Fig. 3). CK13 was detected in 63 of the 108 leukoplakias (58.3 %). CK13 was detected in 11 of the 34 dysplastic leukoplakias (32.4 %) and 52 of the 74 hyperplastic leukoplakias (70.3 %). The “strong” cases were not observed in dysplastic leukoplakias (0 %); on the other hand, the “strong” cases were 23 of the 74 hyperplastic leukoplakias (31.1 %). CK13 was significantly expressed in hyperplastic leukoplakia compared to dysplastic leukoplakia (*p* < 0.01, chi-square test; Table 4).

**Fig. 4:**
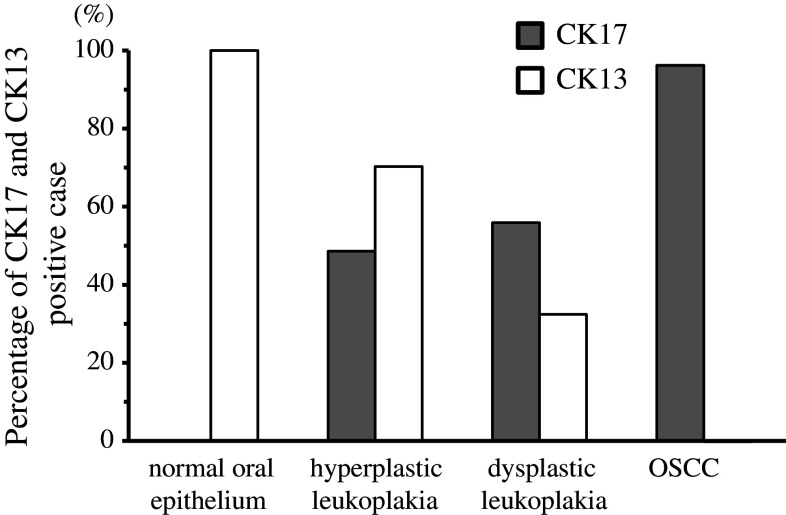
Percentage of CK17- and CK13-positive cases in normal oral epithelium, leukoplakia, and OSCC. The percentages of CK17-positive cases were 0/10 (0 %), 35/74 (48.6 %), 18/34 (54.5 %), and 101/105 (96.2 %), respectively, while the percentages of CK13-positive cases were 10/10 (100 %), 52/74 (70.3 %), 11/34 (32.4 %), and 3/105 (2.9 %), respectively, in normal oral epithelium, hyperplastic leukoplakia, dysplastic leukoplakia, and OSCC

In the comparison between the percentage of CK17-positive cases and that of CK13-positive cases in normal oral epithelium, hyperplastic leukoplakia, dysplastic leukoplakia, and OSCC, that of CK17 was 0/10 (0 %), 35/74 (48.6 %), 18/34 (54.5 %), and 101/105 (96.2 %), respectively, while that of CK13 was 10/10 (100 %), 52/74 (70.3 %), 11/34 (32.4 %), and 3/105 (2.9 %), respectively. Indeed, the percentage of CK17-positive cases elevated gradually in accordance with dysplastic leukoplakia and OSCC, while the percentage of CK13-positive cases declined gradually in accordance with dysplastic leukoplakia and OSCC (Fig. 4). Moreover, dysplastic leukoplakias were observed in 8 of the 11 cases of CK17 “strong” and CK13 “weak”. In contrast, hyperplastic leukoplakias were observed in all 23 CK17 “weak” and CK13 “strong” cases (*p* < 0.01, chi-square test; Table 5).

**Table 5:** Correlation between CK17 and CK13 expression in 34 patients with leukoplakia

	Dysplasia^a^	Non-dysplasia	Total	Significance^b^
CK17 “strong”^c^/CK13 “weak”	8	3	11	
CKlT “weak”/CK13 “strong”	0	23	23	*p* < 0.01

^a^The histological degree of dysplasia was determined according to the criteria of WHO

^b^Statistical significance of the association was determined by the chi-square test

^c^CK17 and CK13 were detected by immunohistochemical staining, and the degree of expression was estimated as described in the “Materials and Methods”

**Fig. 5:**
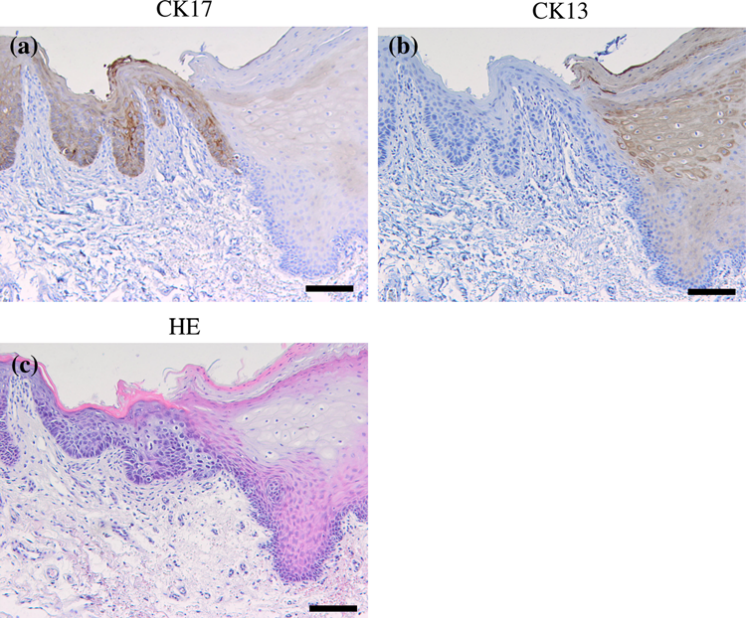
Immunohistochemical staining of CK17 and CK13 in the border between dysplastic leukoplakia and hyperplastic leukoplakia. CK17 was clearly expressed in dysplastic leukoplakia, while it was disappeared in hyperplastic leukoplakia (**a**). CK13 was disappeared in dysplastic leukoplakia, while it was clearly expressed in hyperplastic leukoplakia (**b**). A reciprocal expression of CK17 and CK13 could be found between the two different lesions (original magnification×100, *scale bars* 100 μm)

Figure 5 shows a representative case with the clear border in the leukoplakia due to the expression of CK17 and CK13. CK17 was clearly expressed in dysplastic leukoplakia, while CK13 was disappeared. On the other hand, CK17 was absent in hyperplastic leukoplakia, while CK13 was clearly expressed. These reciprocal expressions of CK17 and CK13 were obvious at the interface between dysplastic and hyperplastic leukoplakia.

### Expression of CK17 mRNA in OSCC cell lines

**Fig. 6:**
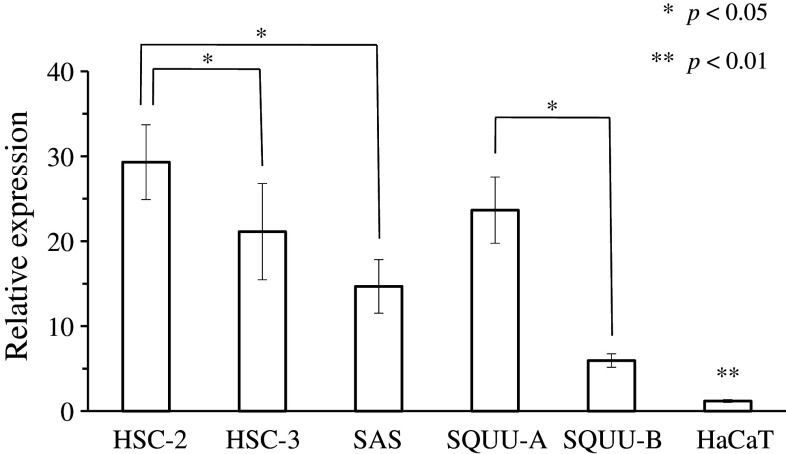
Relative expression of CK17 mRNA in OSCC cell lines and human epithelium cell line. The relative expression of CK17 mRNA in HSC-2 was significantly higher than in HSC-3 and SAS. Moreover, the relative expression of CK17 mRNA in SQUU-A was significantly higher than in SQUU-B. The relative expression of CK17 mRNA in HaCaT was significantly lower than the others. Statistical analyses were performed by Student’s *t*-test

The relative expression of CK17 mRNA in HSC-2 (RQ; 29.3 ± 4.4) was significantly higher than in HSC-3 (RQ; 21.1 ± 5.7) and SAS (RQ; 14.7 ± 3.2) (*p* < 0.05, Student’s *t*-test; Fig. 6). Moreover, the relative expression of CK17 mRNA in SQUU-A (RQ; 23.7 ± 3.9) was significantly higher than in SQUU-B (RQ; 6.0 ± 0.8) (*p* < 0.05, Student’s *t*-test; Fig. 6). The relative expression of CK17 mRNA in HaCaT was significantly lower than in the others (RQ; 1.1 ± 0.2) (*p* < 0.01, Student’s *t*-test; Fig. 6).

## Discussion

The carcinogenesis mechanism of OSCC is very complex, and a wider range of CKs is expressed in OSCC (Wetzels et al. 1992). CK17 may play an important role in the diagnosis of OSCC, since several studies have reported that the over-expression of CK17 could be detected in malignant tissues compared to normal tissues in squamous cell carcinoma of lung (Wetzels et al. 1992), cervix (Carrilho et al. 2004; Ikeda et al. 2008), larynx (Cohen-Kerem et al. 2004), and esophagus (Takahashi et al. 1995; Luo et al. 2004).

Our immunohistochemical results indicate the significant percentage of CK17 expression in OSCC. This is in harmony with the detection of CK17 in breast carcinoma by immunohistochemical staining and microarray analyses (van de Rijn et al. 2002). Moreover, CK17 mRNA is significantly over-expressed compared to CK19 mRNA and CK20 mRNA in OSCC (Toyoshima et al. 2009). Furthermore, up-regulation of CK17 mRNA is observed by microarray analyses in OSCC compared to normal oral epithelium (Ye et al. 2008). In the view of these reports, CK17 is markedly expressed in malignant tissue and thus could be a diagnostic marker of OSCC. The results of CK17 indicate the significant expression in well-differentiated OSCC and the decreasing expression in moderately and poorly differentiated OSCC. This is in disharmony with the CK17 expression in the most of all OSCCs despite clinicopathological variables, considered as a discriminating marker of OSCC (Wei et al. 2009). But the number of cases was too small for a statistical evaluation, since only 30 OSCCs were examined in the study. Therefore, CK17 could be a precise candidate for a diagnostic marker of well-differentiated OSCC.

Focusing on the differentiation of OSCC cell individual, our results show that CK17 is not expressed in the outer layer of cancer nest in well-differentiated OSCC and infiltrating OSCC cells in poorly differentiated OSCC. This is in line with the absence of CK17 expression in the basal area of carcinoma in situ and the outer layer of cancer nest in OSCC (Mikami et al. 2011). It has been suggested that the outer layer of cancer nest is considered as poorly differentiated keratinocyte, since this area is the counterpart of basal and parabasal cells in normal oral epithelium (Bloor et al. 2000; Smedts et al. 1992). In addition, since poorly differentiated OSCC has few well-differentiated keratinocytes and poorly differentiated keratinocytes are observed at the invasive front (Willen et al. 1975), infiltrating OSCC cells could be also considered as poorly differentiated keratinocyte. Therefore, CK17 could be considered as a specific marker of well-differentiated OSCC cells. Since poorly differentiated OSCC has poor outcome because of high malignancy (Pekkola-Heino et al. 1991), it might be possible that CK17 is not strongly expressed in high malignancy of OSCC.

Other immunohistochemical results indicate the strong expression of CK17 in mildly, moderately, and severely dysplastic leukoplakia. This conflicts with the absence of CK17 in mildly and moderately dysplastic leukoplakia (Mikami et al. 2011), but no severely dysplastic leukoplakia was examined in the study. On the other side, our results are compatible with the expression of CK17 mRNA in severely dysplastic leukoplakia (Ohkura et al. 2005). Moreover, CK17 is expressed in mildly, moderately, severely dysplasia of uterine cervix (Martens et al. 2004). Indeed, a marker of the premalignant lesion is important to predict the malignant transformation. Nevertheless, CK17 is expressed in a portion of dysplastic leukoplakias and thus not enough to diagnose individually premalignant lesion. In contrast, CK13 is coordinately expressed in normal oral epithelium and disappeared consistently in dysplastic leukoplakia and OSCC in the view of our results. Therefore, combination of CK17 and CK13 might be a useful marker to diagnose premalignant lesion with high potential of transformation. In addition, over-expression of CK17 and absence of CK13 might be associated with malignant transformation. OSCC tissues change their histological characters and express various CKs (Wetzels et al. 1992). These pathological changes involve the terminally differentiating cell compartment, but it is still unclear about correlation between the changes and the gene expressions of CKs (Debus et al. 1984; Morgan et al. 1987; Vigneswaran et al. 1989; Vaidya et al. 1989). Further studies are needed to clarify what operates to CKs genes.

Real-time RT-PCR results indicate that the quantity of CK17 mRNA in HSC-2 derived from well-differentiated OSCC is significantly higher than that in HSC-3 and SAS derived from poorly differentiated OSCC. This conflicts with the detection of CK17 mRNA in OSCC cell lines by real-time RT-PCR despite their differentiation (Ohkura et al. 2005). In fact, CK17 is synthesized at the stage of cell fate specification within developing skin epithelium (McGowan et al. 2002). Moreover, onset of CK17 expression reflects the determination of the lineage in skin epithelium (McGowan and Coulombe 1998). Furthermore, CK17 is not expressed in the basal cell, while it is expressed in the suprabasal cells that have been already differentiated. This indicates that there is a difference of CK17 expression pattern according to the cell differentiation.

CK17 mRNA quantity was significantly higher in SQUU-A than in SQUU-B, though both are derived from well-differentiated OSCC. The in vitro formation of intermediate filaments is prominently observed in SQUU-A compared to SQUU-B (Morifuji et al. 2000). Furthermore, piling up of SQUU-A cells was partially seen, whereas SQUU-B cells developed multiple layers throughout. Combined with the ability of muscle invasion and cervical lymph node metastasis in vivo, SQUU-B resembles to HSC-3 closely, because HSC-3 also has the ability (Kawahara et al. 1995). Therefore, SQUU-B might have feature of poorly differentiated OSCC compared to SQUU-A.

In conclusion, CK17 expression could be associated with the differentiation and the malignancy of OSCC. A combination pattern of CK17/CK13 might be a suitable marker of malignant transformation.
